# The prognostic impact of B7-H3 and B7-H4 in head and neck squamous cell carcinoma

**DOI:** 10.1007/s00432-022-04244-2

**Published:** 2022-08-08

**Authors:** Mara Borgmann, Agnes Oetting, Felix Meyer, Nikolaus Möckelmann, Conrad Droste, Clara Marie von Bargen, Christina Möller-Koop, Melanie Witt, Kerstin Borgmann, Kai Rothkamm, Christian Betz, Adrian Münscher, Till Sebastian Clauditz, Thorsten Rieckmann

**Affiliations:** 1 Department of Otorhinolaryngology, University Medical Center Hamburg-Eppendorf, Hamburg, Germany; 2 Department of Otorhinolaryngology, Asklepios Klinik Nord, Hamburg, Germany; 3 Department of Radiotherapy and Radiation Oncology, University Medical Center Hamburg-Eppendorf, Hamburg, Germany; 4 Department of Otorhinolaryngology, Marienkrankenhaus Hamburg, Hamburg, Germany; 5 University Cancer Center Hamburg (UCCH), University Medical Center Hamburg-Eppendorf, Hamburg, Germany; 6 Institute of Pathology, University Medical Center Hamburg-Eppendorf, Hamburg, Germany

**Keywords:** HNSCC, B7-H3, CD276, B7-H4, VCTN1, Tissue microarray (TMA), Prognosis, Biomarker

## Abstract

**Purpose:**

Immune checkpoint inhibition is a therapeutic option in many cancer entities. In head and neck squamous cell carcinoma (HNSCC) targeting of the PD-1/PD-L1 (B7-H1) axis is approved in recurrent/metastatic disease and is being explored in the curative setting. Here, we evaluated two related members of the B7 family, B7-H3 & B7-H4, for their prognostic impact under standard treatment.

**Methods:**

A tissue microarray (TMA) of a single center HNSCC cohort was stained for B7-H3 and B7-H4. Staining intensity and the number of tumor cells stained were assessed, and the expression was scored according to an established algorithm. Staining scores were correlated with clinicopathological parameters and associated with patient survival. mRNA levels of both proteins were associated with patient outcome using the TCGA dataset.

**Results:**

mRNA levels of B7-H3 and B7-H4 were not significantly associated with patient survival. TMA analysis revealed interpretable protein staining in 408 samples. Strong staining was the most frequent category for B7-H3 and no staining for B7-H4. In patients with p16-negative oropharyngeal SCC (OPSCC) and in a pooled cohort consisting of p16-negative OPSCC, laryngeal, hypopharyngeal and oral cavity SCC, strong B7-H3 expression was associated with better overall survival. For the latter cohort, this was in part due to reduced lymph node involvement. B7-H3 expression in p16-positive OPSCC and B7-H4 expression were not associated with outcome.

**Conclusion:**

Despite a possible role in tumor immune escape, B7-H3 was associated with favorable prognosis in HPV-negative HNSCC in our cohort. The underlying mechanisms and a potential impact for B7-H3 targeting remain to be elucidated.

**Supplementary Information:**

The online version contains supplementary material available at 10.1007/s00432-022-04244-2.

## Introduction

Head and neck cancer represents the sixth most common human malignancy, with approximately 450,000 new cases of head and neck squamous cell carcinoma (HNSCC) diagnosed annually worldwide (Cohen et al. 2018; Zhuang and Xu 2020). At time of diagnosis most patients present with advanced stage disease. Outcome is favorable in early-stage disease with 5-year survival rates ranging from 75 to 90%. However, most patients present with either locally advanced disease with a five-year survival rate of approximately 50% (Du et al. 2019). Standard curative treatment consists of surgery and/or (chemo)radiation. The choice of primary treatment in part depends on the geographical region, and multimodal treatment is common in advanced stages. The only accepted prognostic biomarker is the presence of human papillomavirus (HPV) or the surrogate marker p16 in oropharyngeal tumors, where they confer a decidedly favorable prognosis, which has led to an adjustment in the TNM staging system (Huang and O'Sullivan 2017).

An effective way to improve therapeutic efficacy for HNSCC, as in many other tumor entities, is to block immune checkpoint receptors (Robert 2020). A multitude of studies have shown that T-lymphocytes, as part of the adaptive immune system, play a central role in anti-tumor immunity (Mami-Chouaib et al. 2018; St Paul and Ohashi 2020; Waldman et al. 2020). The activity status of T-lymphocytes as well as other immune cells, such as tumor-associated macrophages and NK cells, is regulated by a variety of immune modulating receptors, including immune checkpoint receptors, which play critical roles in the induction and maintenance of immune tolerance to normal tissue. The B7 immunoglobulin superfamily contains both, immune stimulators and potent immune checkpoint receptors including the well-examined representatives PD-L1 and PD-L2 (B7-H1, B7-DC) (Pardoll 2012). Inhibition of the PD-1/PD-L1 axis with or without chemotherapy has been shown to improve overall survival (OS) in first-line treatment of PD-L1-expressing recurrent/metastatic HNSCC as compared to the chemotherapy plus cetuximab-based EXTREME regimen (Burtness et al. 2019). However, despite these recent advances, the response rates towards immune checkpoint inhibition in HNSCC are far from being satisfactory (Ferris et al. 2018; Burtness et al. 2019).

Further immunoinhibitory members of the B7 superfamily, which, similar to PD-L1, are expressed on the surface of a variety of normal tissue but also tumor cells, include B7-H3 (= CD276) and B7-H4 (= VCTN1). In an oncological context, high expression of B7-H3 has been reported in several cancer types, including melanoma, glioma, breast and ovarian cancer, hepatocellular, colorectal, pancreatic, and non-small cell lung carcinoma (NSCLC). High expression of B7-H3 has been repeatedly described as a negative prognostic factor, for example in pancreatic, colon, hepatocellular, ovarian or breast cancer (Ingebrigtsen et al. 2012; Xu et al. 2016; Zheng et al. 2019). B7-H4 was reported to be expressed in various tumor types, such as spleen, lung, thymus, ovarian, breast, melanoma, gastric and renal cell carcinoma, NSCLC, esophageal, endometrial cancer, colorectal and pancreatic cancer and has mostly been associated with worse clinical outcome across various cancer types (Xu et al. 2016; Meng et al. 2017; Ding et al. 2021).

For HNSCC, the role of both proteins for tumor progression and patient survival has not yet been conclusively demonstrated. Katayama et al. investigated the expression of B7-H3 in 37 samples from hypopharyngeal squamous cell carcinoma (HSCC) patients and observed a higher rate of distant metastasis after tumor-free periods in patients whose tumors showed higher B7-H3 expression (Katayama et al. 2011). Hu et al. reported high expression in 66.1% of HNSCC specimens and a correlation with poor survival in a cohort of 274 tumor samples (Hu et al. 2019) and Li et al. similarly reported of inferior survival upon high expression in a cohort of 122 patients with laryngeal SCC (LSCC) (Li et al. 2021). High expression levels of B7-H3 were associated with reduced T-cell infiltration (Katayama et al. 2011; Li et al. 2021). Recently, it has further been shown that expression of B7-H3 and subsequent immune evasion in HNSCC is regulated by a long non-coding RNA (LINC01123) through repression of miRNA(miR-214-3p)-mediated inhibition of B7-H3 expression (Li et al. 2022) and that especially HNSCC cancer stem cells (CSCs) utilize B7-H3 to evade immune surveillance. Anti-B7-H3 antibodies were shown to eliminate such CSCs in a CD8^+^ T cell-dependent manner in an HNSCC mouse model (Wang et al. 2021). For B7-H4 there are only limited data regarding its potential prognostic impact in HNSCC. Wu et al. reported that in oral SCC, B7-H4 expression was associated with lymph node status and pathological grade. High expression was associated with poor overall survival (Wu et al. 2016). In LSCC co-expression of B7-H4 with markers of epithelial mesenchymal transition (EMT) was described, and overexpression of B7-H4 induced their expression in LSCC cells, suggesting a causative relationship (Chen et al. 2017).

In summary, B7-H3 and B7-H4 have both been described as prognostic markers in head and neck cancer but to date the data are sparse and often only related to specific subsites. As immunoregulatory factors, both proteins represent potential targets for immune checkpoint inhibition as an alternative to, or in combination with, current PD-1/PD-L1 targeting approaches. The anti-B7-H3 antibody enoblituzumab and the anti-B7-H4 antibody FPA150 are already being tested in clinical trials. Since data on B7-H3 and B7-H4 expression patterns in HNSCC are still limited, mostly relate to small patient cohorts, and have not been studied together, we investigated the impact of both markers on patient survival in a retrospective single center patient cohort in a tissue microarray (TMA) format and the TCGA dataset.

## Materials and methods

### Clinical in silico analysis

Clinical- and mRNA expression data for B7-H3 or B7-H4 were extracted from the HNSCC TCGA cohort of the cBioPortal (http://cbioportal.org) (Cerami et al. 2012; Gao et al. 2013). For each tumor (*n* = 516), B7-H3 or B7-H4 gene expression was analyzed and the 25% of tumors with the highest and lowest gene expression were compared for overall patient survival. *p*-values were calculated using log-rank test.

### Specimen collection and TMA construction

Survival and the clinicopathological data from HNSCC patients treated with curative intent at the University Medical Center Hamburg-Eppendorf were analyzed retrospectively. Tumors were diagnosed between 1992 and 2013, with a vast majority of 95% diagnosed between 2000 and 2013. Tissue samples were either obtained from primary tumors during surgical resection or from diagnostic biopsies. The use of archived diagnostic leftover tissues and their analysis for research purposes as well as patient data analysis have been approved by local laws (HmbKHG,§12,1) and by the local ethics committee (Ethics commission Hamburg, WF-049/09). The study has been carried out in compliance with the Helsinki Declaration.

Tissue samples utilized for TMA construction had been fixed in buffered 4% formalin and embedded in paraffin. TMA construction was performed as previously described (Bubendorf et al. 1999; Dancau et al. 2016). In brief, hematoxylin–eosin-stained sections were made from each selected primary tumor block to identify representative tumor regions. A tissue cylinder of 0.6 mm diameter was punched from each block using a homemade semi-automated tissue arrayer. For immunohistochemical staining, three-micrometer TMA sections were prepared using the Paraffin Sectioning Aid System (Instrumentics, Hackensack, NJ, USA).

### Immunohistochemistry

Freshly cut TMA sections were analyzed in a single experiment in one day. After 10 min of peroxidase blocking with H2O2 (DAKO S2023) B7-H3 (monoclonal rabbit antibody; clone SP265; Zytomed) and B7-H4 (monoclonal mouse antibody, clone H74; Antibodies Online) antibodies were used for immunohistochemical staining. High temperature pretreatment of the slides was conducted in an autoclave in citrate buffer, pH 7.8, for 5 min. The Envision system (DAKO 5007) was used to visualize the immunostaining. The staining was assessed separately by two experienced pathologists (TSC & CMvB) using a well-established scoring system based on staining intensity (0, 1, 2, 3—referring to absent, low, intermediate or high intensity) and the fraction of tumor cells stained (Simon et al. 2010; Steurer et al. 2021). The final IHC score (negative, weak, moderate, strong) is built from these parameters as follows: negative scores had a staining intensity of 0; weak scores had a staining intensity of 1 + in ≤ 70% of tumor cells or a staining intensity of 2 + in ≤ 30% of tumor cells; moderate scores had a staining intensity of 1 + in > 70% of tumor cells, a staining intensity of 2 + in > 30% and ≤ 70% of tumor cells or a staining intensity of 3 in ≤ 30% of tumor cells; and strong scores had a staining intensity of 2 + in > 70% of tumor cells or a staining intensity of 3 in > 30% of tumor cells. In the event of discrepancies, tissue spots were re-analyzed by both pathologists and a consensus result was assigned.

### Analysis of patient survival

Statistical analysis was performed using R and Bioconductor environment (Huber et al. 2015) for data processing, analysis and evaluation. Survival analyses were performed according to the Kaplan–Meier method and the Log-rank test using the R packages “survival” and “survminer”, multivariate analyses were performed fitting a Cox proportional hazards regression model using the “survival” package (Therneau and Grambsch 2000; Kassambara and Kosinski 2018). Potential associations between variables were tested using the Pearson correlation co-efficient (R-packages: reshape and corrplot, Wickham 2007; Wei and Simko 2017). All statistical analyses are to be considered exploratory. The reported *p*-values are two-sided and used as descriptive measures only.

### Further data analyses

Depiction of protein expression scores were performed using GraphPad Prism 6*.*

## Results

### No clear association of B7-H3 and B7-H4 mRNA levels and patient survival

**Fig. 1:**
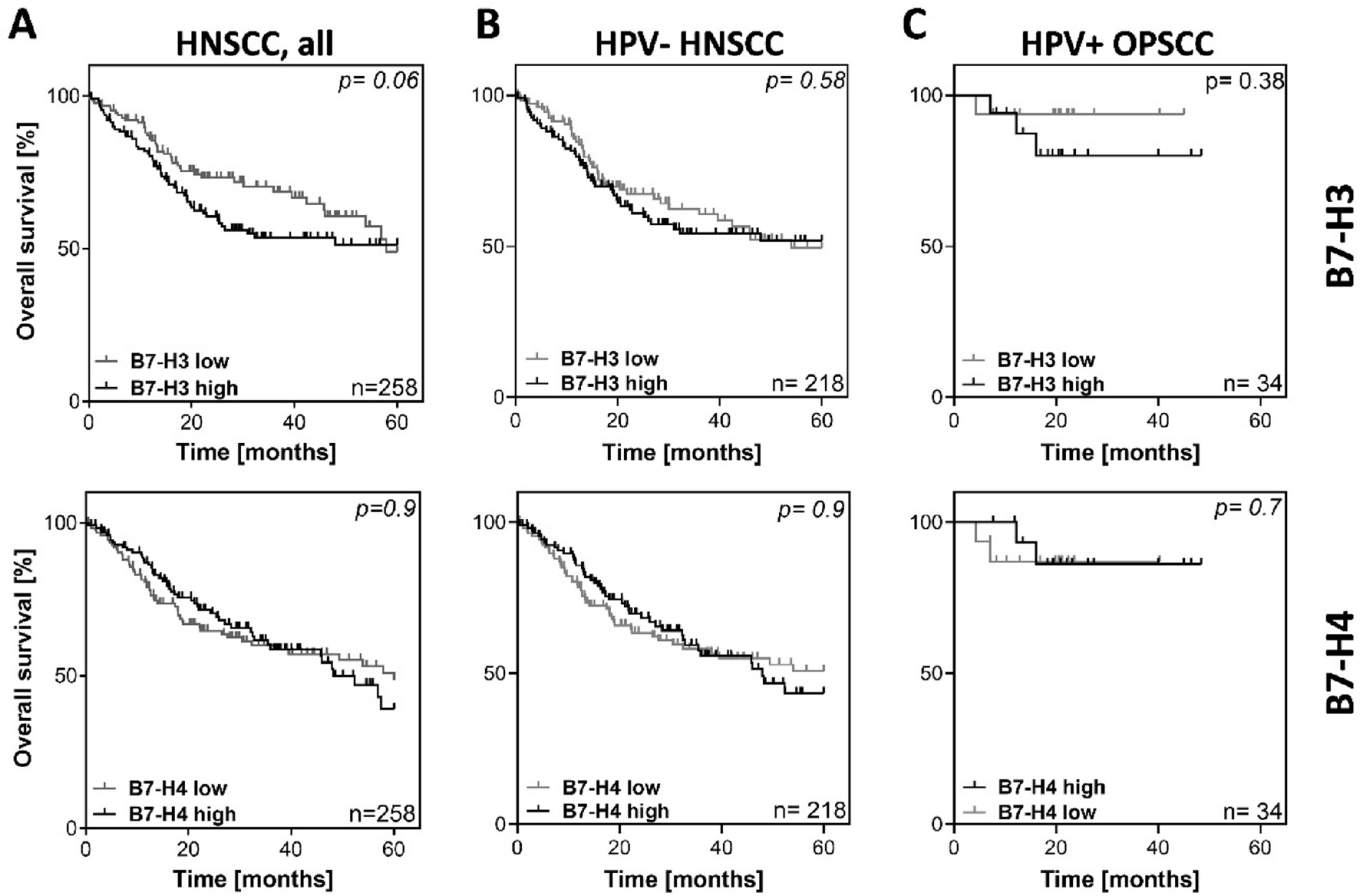
Overall survival of patients in the HNSCC TCGA cohort with respect to B7-H3 and B7-H4 mRNA expression. Depicted are the upper (high) and lower (low) quartiles of cases based on the mRNA expression levels of the respective proteins in the TCGA HNSCC cohort. **A** Whole cohort. **B** HPV- cohort. **C** HPV+ OPSCC only. p-values were calculated using log-rank test

To investigate the effect of mRNA expression of B7-H3 and B7-H4 on survival in HNSCC, the expression of both markers was analyzed using the TCGA dataset (cBioportal.com). Figure. 1 depicts overall survival (OS) in dependence of B7-H3 and B7-H4 expression based on the comparison of the lower and upper quartiles. High mRNA expression of B7-H3 showed a trend towards a negative prognostic impact on OS in the whole cohort but the difference failed to reach significance (*p* = 0.06) (Fig. 1A, top). No association or trend was observable for B7-H4 mRNA expression (*p* = 0.9) (Fig. 1A, bottom). Due to the different biology of HPV-positive (HPV+) HNSCC, we next excluded all annotated HPV+ cases and the few OPSCC with unknown HPV/p16 status from the analysis. No clear differences were observed in the remaining HPV- tumors (Fig. 1B) or in a cohort consisting only of HPV+ OPSCC, although, due to the low numbers of cases and events, the latter results should be interpreted with caution (Fig. 1C).

### High B7-H3 and low B7-H4 protein expression in HNSCC

To assess the impact of B7-H3 and B7-H4 protein expression on patient survival in HNSCC, the expression of both proteins was analyzed in a retrospective cohort in a tissue micro array (TMA) format. All patients had been treated with curative intent at the University Medical Center Hamburg-Eppendorf. Overall, 398 and 395 samples showed interpretable staining for B7-H3 and B7-H4, respectively. OPSCC was the most frequent sub-entity (185), followed by LSCC (140), HSCC (51), and oral cavity tumors (23). An overview of the clinicopathological characteristics of the patients and tumors is provided in Table 1.

**Table 1:** Clinicopathological characteristics of patient samples

Patient characteristics				
Interpretable staining, number (%)				
B7-H3 and/or B7-H4			408 (100)	
B7-H3			398 (97.5)	
B7-H4			395 (96.8)	
Age, median (range)			61 (31–85)	
Sex, number (%)				
Male			316 (77.5)	
Female			90 (22.05)	
n.a			2 (0.5)	
Location, number (%)				
Oropharynx			185 (45.3)	
p16+ (% of OPSCC)			68 (36.8)	
p16- (% of OPSCC)			93 (50.3)	
p16 n.a. (% of OPSCC)			24 (13)	
Larynx			140 (34.3)	
Hypopharynx			51 (12.5)	
Oral cavity			23 (5.6)	
Nasopharynx			6 (1.5)	
n.a			3 (0.8)	
T classification, number (%)				
T1			97 (23.8)	
T2			112 (27.5)	
T3			94 (23)	
T4			97 (23.8)	
n.a			8 (1.9)	
N classification, number (%)				
N0			184 (45.1)	
N1			58 (14.2)	
N2			141 (34.6)	
N3			18 (4.4)	
n.a			7 (1.7)	
Therapy, number (%)				
Surgery			135 (33.1)	
Surgery + (chemo)radiation			189 (46.3)	
Chemoradiation			50 (12.3)	
Radiotherapy			10 (2.5)	
Other			12 (2.9)	
n.a			12 (2.9)	

T- and N-classification were performed according to the 7th edition of the Union for International Cancer Control (UICC). Classification represents clinical staging for tumors treated with definitive (chemo)radiation and pathological staging for resected tumors. For 24 spots of oropharyngeal tumors, p16 status was not available (n.a.) because of indistinct scoring (intermediate or high staining intensity in ≥ 40 but ˂70% of tumor cells) or because of a lack of tumor tissue in the p16-stained tissue microarray section

**Fig. 2:**
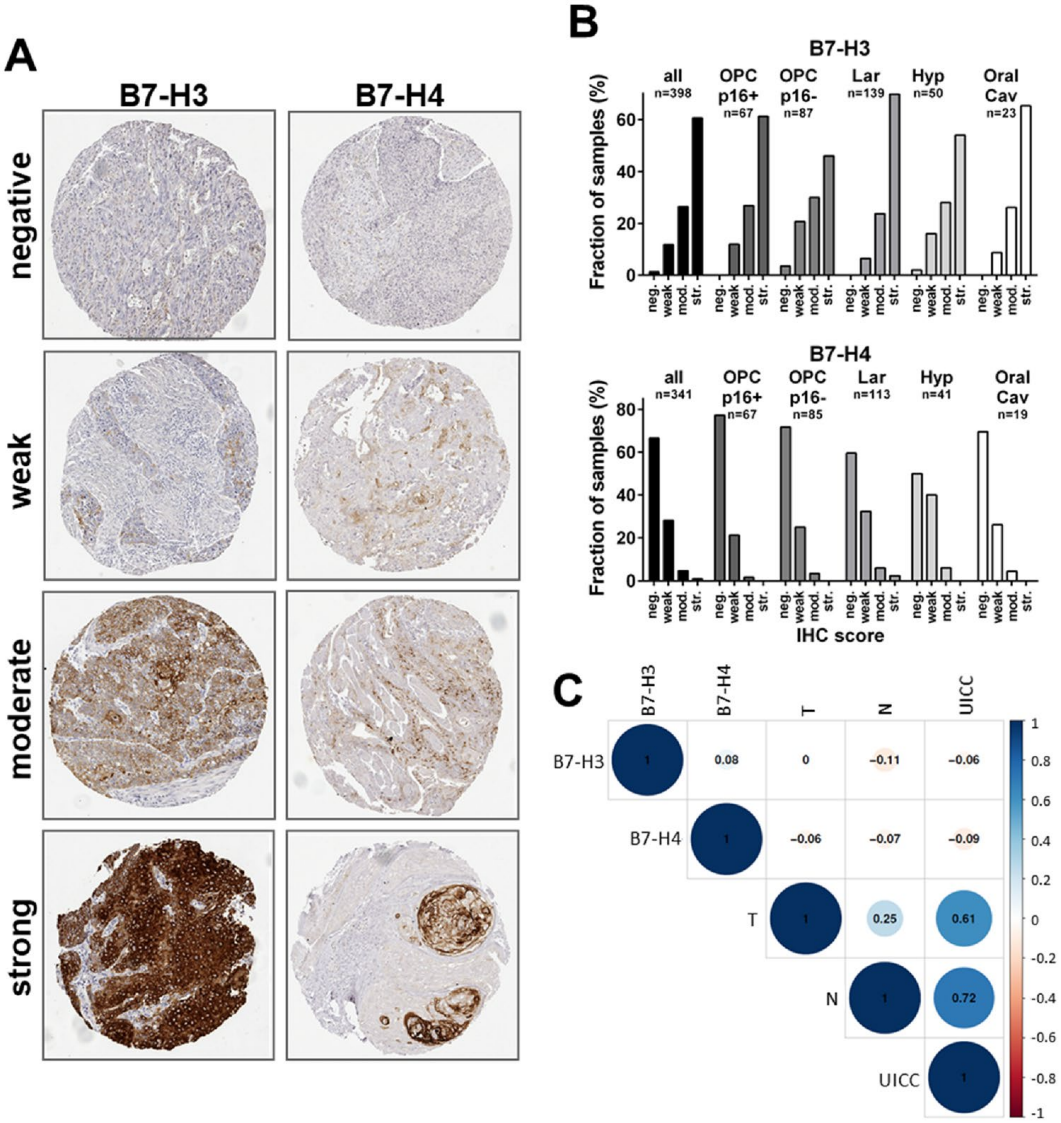
Immunohistochemical staining and correlation analysis of B7-H3 and B7-H4. **A** Staining examples. **B** Distribution of staining categories in all HNSCC and the indicated main sublocations. neg.: negative; mod.: moderate; str.: strong. **C** Whole cohort correlation analysis does not demonstrate an association of protein expression with each other, T- or N-stage. Correlation of T- & N- with UICC-stage (7th edition) serves as an internal control

In an established semiquantitative analysis, the immunohistochemical staining intensities of B7-H3 and B7-H4 were scored as 0, 1, 2 or 4 and the respective percentage of tumor cells was estimated (Fig. 2A). The final IHC score was built from both parameters with individual spots being categorized as *negative*, *weak*, *moderate* or *strong* (see “Materials and methods” for details). The staining of both proteins was mostly restricted to the plasma membrane and cytoplasm. Overall, a major difference in the expression of both proteins was observed. In the whole cohort and in each of the respective sublocations, the highest staining category *strong* was the most frequent one for B7-H3, whereas *negative* staining was the most frequent category for B7H4 (Fig. 2B). Normal tissue control spots of the TMA demonstrated far weaker B7-H3 expression as compared to the tumor spots. Only 3 of the overall 50 samples showed strong B7-H3 staining, while a complete lack of staining was observed in 29 samples. For B7-H4, expression was moderately enhanced in the normal tissue vs. the tumor samples with *weak* staining being the most frequent category (Supplementary Fig. 1). So while strong expression of B7-H3 is highly frequent in HNSCC and considerable expression of B7-H4 a rather rare event, we further assessed whether protein expression levels were associated with each other or with T-, or N-stage. Correlation analyses revealed no clear associations for any of the two immune checkpoint receptors (Fig. 2C). To account for the different biology of HPV+ and HPV- HNSCC also in the TMA analyses, we additionally performed separate analyses in OPSCC positive for the HPV surrogate marker p16 (p16+ OPSCC) and in a pooled cohort consisting of p16-negative (p16-) OPSCC plus all laryngeal, hypopharyngeal and oral cavity carcinomas, in which HPV-driven cases are known to constitute only a minor fraction of less than 5% when assessing HPV E6/E7 mRNA in addition to p16 and/or HPV DNA (Castellsague et al. 2016; Taberna et al. 2016; Wittekindt et al. 2017; Tagliabue et al. 2020; Nauta et al. 2021; Simoens et al. 2021). Again, the analyses revealed no clear associations (Supplementary Fig. 2).

### Associations of B7-H3 and B7-H4 protein expression levels and patient survival

To address the impact of B7-H3 and B7-H4 protein expression on patient survival, we dichotomized the staining scores according to the most abundant categories, which means *strong* vs. *all others* for B7-H3 and *negative* vs. *all others* for B7-H4.

**Fig. 3:**
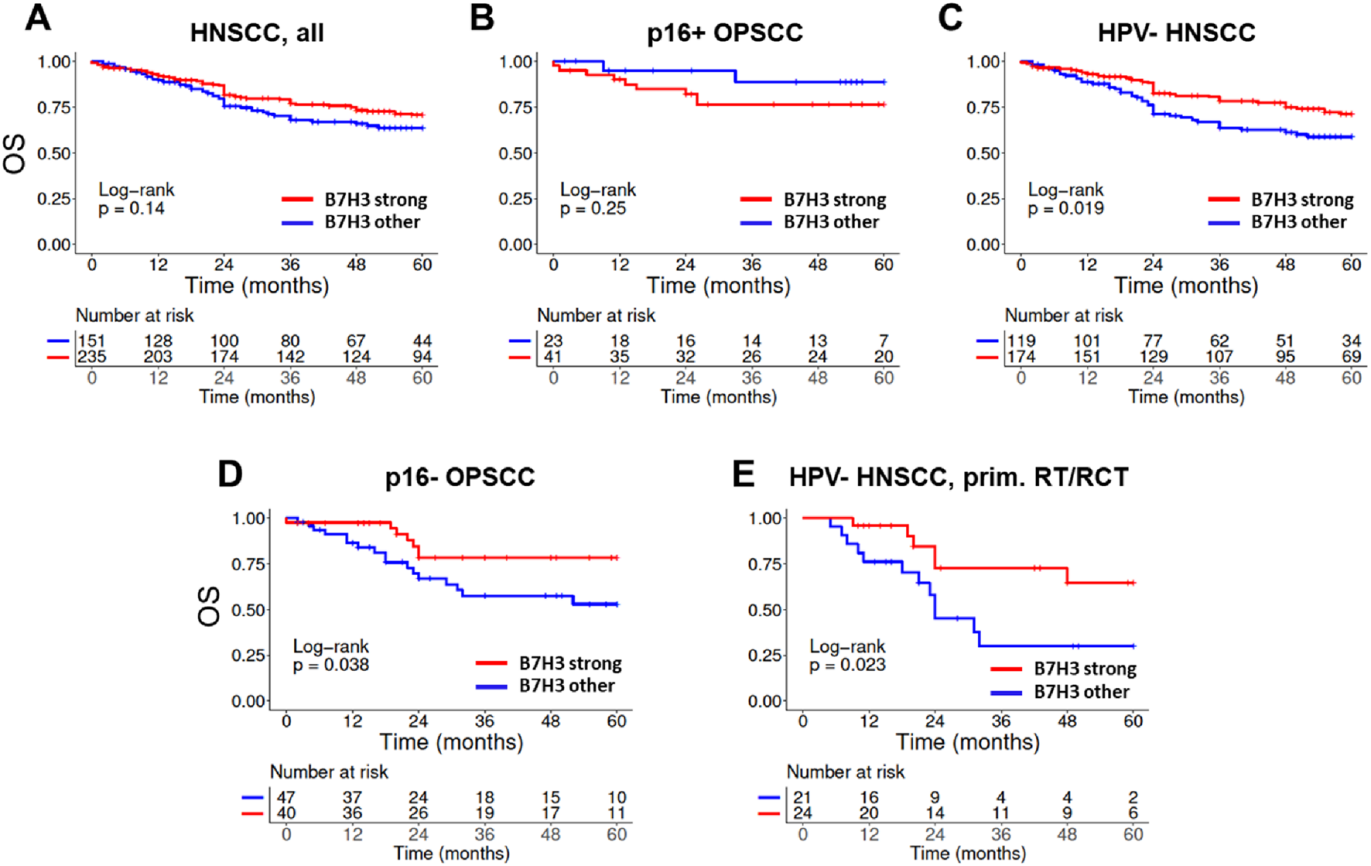
Association of patient overall survival and B7-H3 expression. B7-H3 expression was categorized as either strong or other, which includes all samples scored as negative, weak or moderate. **A** All patients. **B** Patients with p16+ OPSCC. **C** Patients with HPV- HNSCC and **D** the subfraction of patients with p16- OPSCC. **E** Patients with HPV- HNSCC treated with primary radio(chemo)therapy

In case of B7-H3, no significant association of protein expression and overall survival (OS) was observed for the whole cohort (Fig. 3A). In separate analyses for p16+ OPSCC and the above-described pooled cohort of largely HPV-negative tumors (in the following referred to as HPV-negative (HPV-) HNSCC), we observed a clear difference for the impact of B7-H3 regarding OS. In patients with p16+ OPSCC, those with tumors demonstrating strong B7-H3 expression showed a trend towards inferior survival but the difference did not reach significance (Fig. 3B). In contrast, patients with HPV- HNSCC showing strong B7-H3 expression demonstrated significantly favorable OS (*p* = 0.019) (Fig. 3C). When looking at the individual sublocations of the pooled HPV- cohort, significantly better OS was observed for p16- OPSCC (*p* = 0.038) (Fig. 3D) and a trend was observed in laryngeal SCC but not in hypopharyngeal tumors or in the rather few cases of oral cavity tumors in our cohort (Supplementary Fig. 3A). Regarding the interplay of B7-H3 expression and patient treatment, significantly favorable survival upon strong staining was observed after primary (chemo)radiation (RT/RCT) in the whole HPV- cohort (*p* = 0.023) (Fig. 3 E), which was largely based on profound differences in laryngeal tumors and p16- OPSCC (Supplementary Fig. 3B). It has to be mentioned, however, that the limited numbers of patients treated with primary RT/RCT in these two subgroups urge caution in the interpretation of the data and confirmatory studies are required. Regarding RFS after primary RT/RCT, we observed trends towards favorable outcome in patients with HPV- HNSCC and p16- OPSCC and a significant difference in laryngeal SCC (Supplementary Fig. 4).

**Fig. 4:**
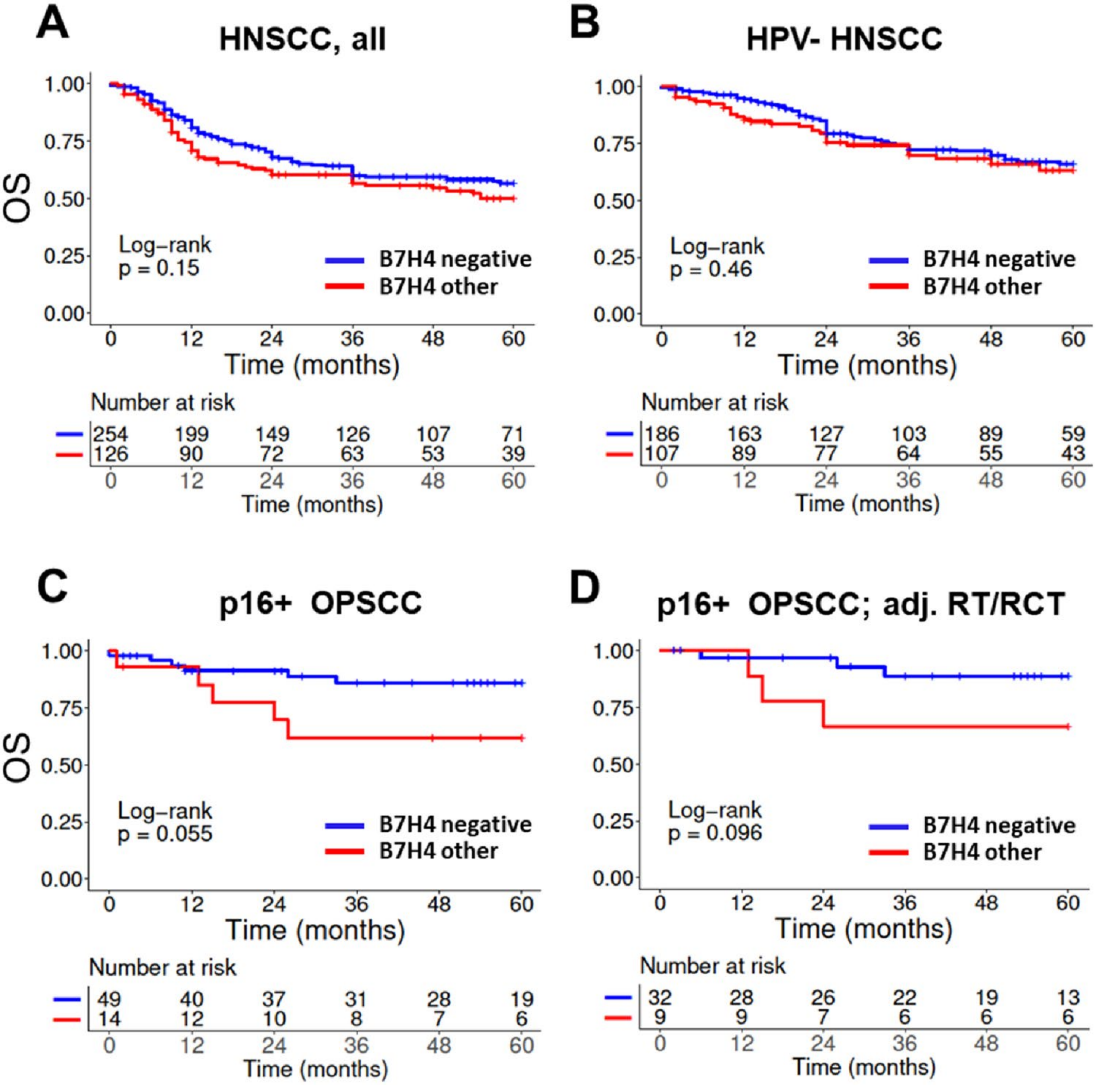
Association of patient overall survival and B7-H4 expression. B7-H4 expression was categorized as either negative or other, which includes all samples showing any kind of B7-H4 staining. **A** All patients. **B** Patients with HPV- OPSCC. **C** Patients with p16+ OPSCC and **D** patients with p16+ OPSCC treated with surgery and adj. RT/RCT

For B7-H4 we also dichotomized the staining scores according to the most abundant category, which in this case means *negative* vs. *all others*. We did not observe statistically different OS in dependence of absence or presence of B7-H4 staining but trends towards inferior survival upon expression in p16+ OPSCC and the biggest treatment group of these tumors, namely patients receiving surgery and adjuvant RT/RCT **(**Fig. 4). Further and bigger cohorts of p16+ OPSCC will be necessary to clarify whether and to what extent B7-H4 expression may represent a prognostic marker in these tumors.

### Multivariable analyses

In a multivariable analysis including expression score, T- and N-stage, sex, and age, significance for strong B7-H3 expression in HPV-negative HNSCC was missed (HR 0.70, *p* = 0.10094) (Table 2). In line with a faint negative association of B7-H3 expression with N- but not T-stage (Supplementary Fig. 2), significance was only retained when N-stage was omitted from the multivariable analyses (not shown). For p16-negative OPSCC, the sublocation demonstrating the strongest prognostic impact of B7-H3, significance was retained in the multivariable analysis also when N-status was included (HR 0.3586, *p* = 0.0303) (Table 2).

**Table 2:** Multivariable analyses

Variables	HR	95% CI	*p*-value
*HPV- HNSCC*			
B7-H3	0.700	0.457–1.072	0.10094
T-stage	1.411	1.145–1.740	** 0.00124
N-stage	1.361	1.079–1.716	** 0.00920
Age	1.469	1.161–1.857	** 0.00134
Sex (m,f)	0.834	0.497–1.402	0.49466
*p16- OPSCC*			
B7-H3	0.3586	0.142–0.907	* 0.0303
T-stage	1.543	1.045–2.279	* 0.0293
N-stage	1.577	1.007–2.470	* 0.0465
Age	1.264	0.832–1.920	0.2731
Sex (m,f)	1.368	0.590–3.169	0.4652

Asterisks indicate significant associations of variables and survival, with * and ** indicating *p* < 0.05 and *p* < 0.01, respectively (Cox proportional hazards regression). The table includes all tested variables of the respective analyses

## Discussion

The prognostic and predictive significance of the B7 immunoglobulin family members B7-H3 and B7-H4 is considerably less characterized compared to the well-known family members PD-L1 and PD-L2. For PD-L1, a recent meta-analysis reports favorable survival especially in patients with HPV+ OPSCC showing high PD-L1 expression also under non-immunotherapy treatment (Polesel et al. 2021). This demonstrates that factors with clearly established roles in immunosuppression can serve as positive prognostic markers under standard regimes outside the context of immunotherapy. For B7-H3 and B7-H4, the counter-receptors for both proteins so far remain elusive, which clearly limits the clarification of their exact molecular functions. B7-H3 was originally described as an immune stimulatory molecule and has been implicated in autoimmune diseases (Chapoval et al. 2001; Luo et al. 2004; Chen et al. 2020). In contrast, recent studies report a role as an immune checkpoint molecule, amongst other functions limiting anti-tumor responses through T- and NK-cells, but also the promotion of non-immunological protumorigenic functions has been reported, such as migration and invasion, angiogenesis or chemoresistance, and effects on tumor metabolism (Kontos et al. 2021; Zhou and Jin 2021).

B7-H3 was reported to be strongly expressed at the tumor cell surface in many cancer entities, as compared to only weak expression in normal tissues. Therefore, B7-H3 was referred to as a pan-cancer antigen, and various preclinical studies and clinical trials have been and are being conducted to exploit B7-H3 as a tumor specific therapeutic target, for example for bispecific antibodies (anti-CD3 x anti-B7-H3) linking immune cells to tumor cells, or for CAR-T cells (Yang et al. 2020; Kontos et al. 2021; Zhou and Jin 2021). A role as target in immune checkpoint inhibition, alone or combined with targeting of the PD1/PD-L1 axis, is also emerging in HNSCC. In this case, immune checkpoint blockade through B7-H3 inhibition as well as antibody-dependent cellular cytotoxicity (ADCC) due to high amounts of antibodies binding to tumor cells with strong B7-H3 expression may both contribute to a meaningful antitumor effect (Aggarwal et al. 2022).

Regarding its prognostic significance in HNSCC, it was mainly observed that B7-H3 is associated with inferior survival and a more advanced stage. However, data for both B7-H3 and even more for B7-H4 are rather sparse. Concerning the expression of B7-H4, our data did not confirm a negative prognostic value in HPV- HNSCC but show a trend toward inferior prognosis in HPV+ OPSCC. However, significance was not reached in our limited cohort and further, and at best larger, p16+ OPSCC cohorts will be necessary to address this issue. For B7-H3, our TMA data clearly confirm high expression in HNSCC tumors, in agreement with previous studies (Hu et al. 2019), with overall slightly more than 60% of samples categorized as *strong*, while mostly *weak* or *absent* staining was observed in the normal tissue control spots (Fig. 2B, Supplementary Fig. 1). However, we did not observe an association of B7-H3 expression with advanced tumor stages or inferior outcome but surprisingly, even observed moderately favorable survival in patients with HPV- disease, whose tumors showed *strong* staining **(**Fig. 3). Multivariable analyses suggest that this favorable survival may be related to a reduced lymph node involvement and that the most robust prognostic impact may exist for p16- OPSCC. In contrast, we did not observe any clear difference regarding patient survival in the HPV- HNSCC sub-cohort in the TCGA dataset based on mRNA expression (Fig. 1). Various explanations for this discrepancy are possible. First, protein levels can be regulated post-transcriptionally and therefore differ from mRNA expression levels. The cutoffs used in both methods are different comparing the upper and lower quartiles, which can be applied independently of the absolute expression levels, in the mRNA analysis versus an immunohistochemistry score in which more than 50% of tumor samples were scored as *strong*. Of note, separation by the median mRNA expression level, which in this comparison may closer resemble the TMA scoring, did not yield more similar results (not shown). A potential disadvantage of IHC analyses is the dependence on the utilized antibodies but especially since the B7-H3 antibody used here is a clone whose specificity had been tested via knockout experiments, we are very confident regarding the quality of our staining. Lastly, the TCGA cohort contains less oropharyngeal and more oral cavity tumors than our TMA cohort and the impact in specific subgroups should clearly be further investigated but requires rather large cohorts. Another important parameter when assessing patient outcome is the type of treatment delivered to the patients. Unfortunately, this information was mostly not provided in previous reports (Katayama et al. 2011; Hu et al. 2019). In a study by Li et al. the inferior outcome of LSCC patients with high B7-H3 expression was observed in a cohort of primarily operated patients (Li et al. 2021). Our results now suggest favorable survival of patients with HPV- tumors showing strong expression, especially when treated with definitive radio(chemo)therapy. However, due to the small numbers of patients and events in such specific subfractions, further analyses in other and, at best, larger cohorts are needed. Whether, and if so to what extent, immune modulatory or immune-independent functions of B7-H3 may contribute to a given enhanced radio(chemo)sensitivity of HPV- HNSCC with strong B7-H3 expression and whether this needs to be considered for potential combination therapy with anti-B7-H3 antibodies is an important question for future research.

## Supplementary Information

Below is the link to the electronic supplementary material.

Supplementary file1 (DOCX 880 kb)
