# Supplementary material for: The prognostic impact of B7-H3 and B7-H4 in head and neck squamous cell carcinoma
Source: J Cancer Res Clin Oncol. 2022 Aug 8;149(7):3383–93. doi: 10.1007/s00432-022-04244-2 (PMC10314856; doi:10.1007/s00432-022-04244-2)
Supplement: Supplementary file 1 — Supplementary file1 (DOCX 880 kb) [file 432_2022_4244_MOESM1_ESM.docx]

**Supplementary Figures**

**
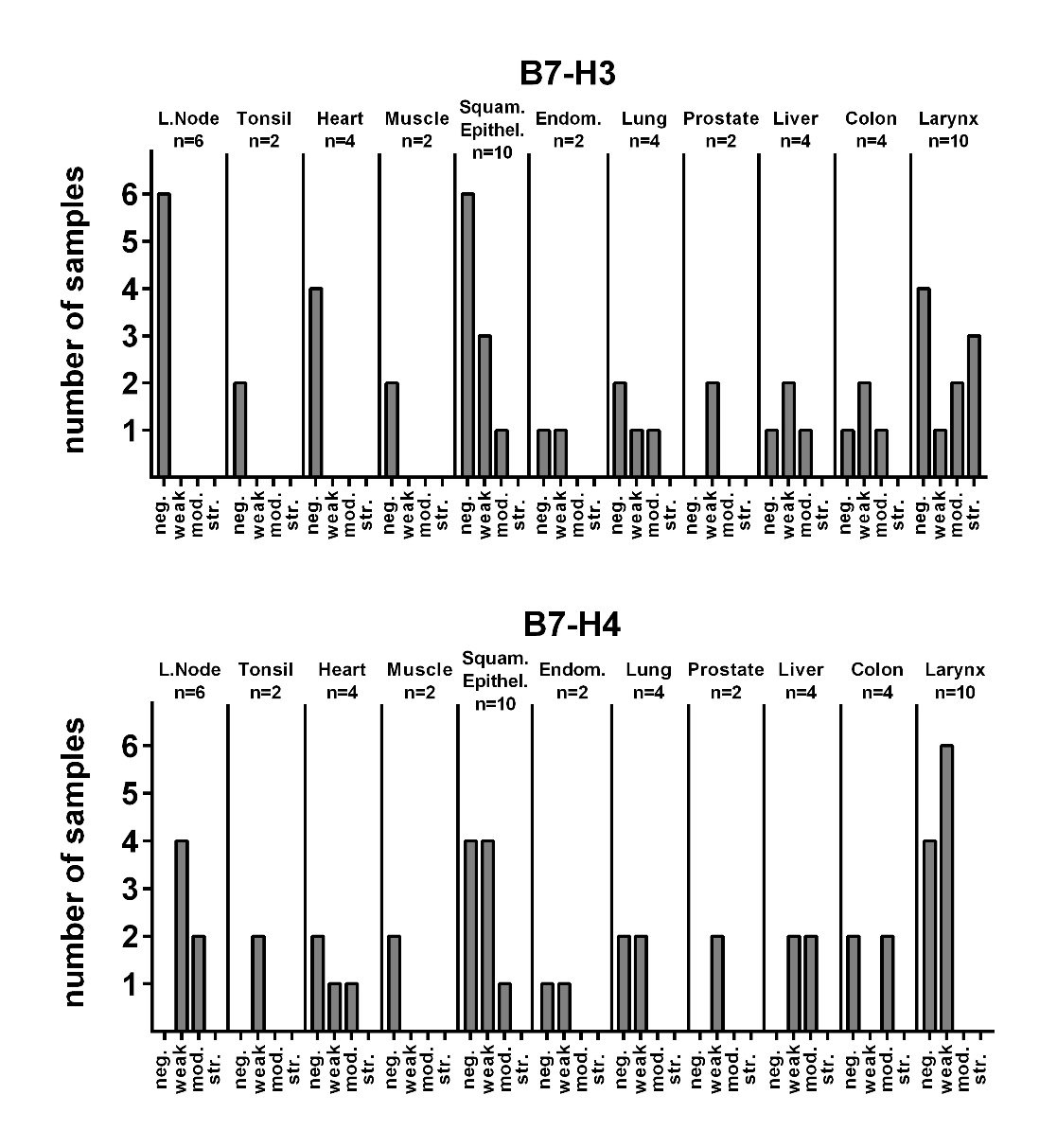
**

**Supplementary Figure 1. Immunohistochemical staining of normal tissue control samples.**

Distribution of staining categories in the normal tissue control samples of the TMA in absolute numbers. Staining of normal tissue for B7-H3 is clearly reduced as compared to the HNSCC tumor samples. Note that the mostly small number of samples per tissue type limits accurate conclusions.

neg.: negative; mod.: moderate; str.: strong; Squam.Epithel.: Squamous epithelium; Endom.: Endometrium


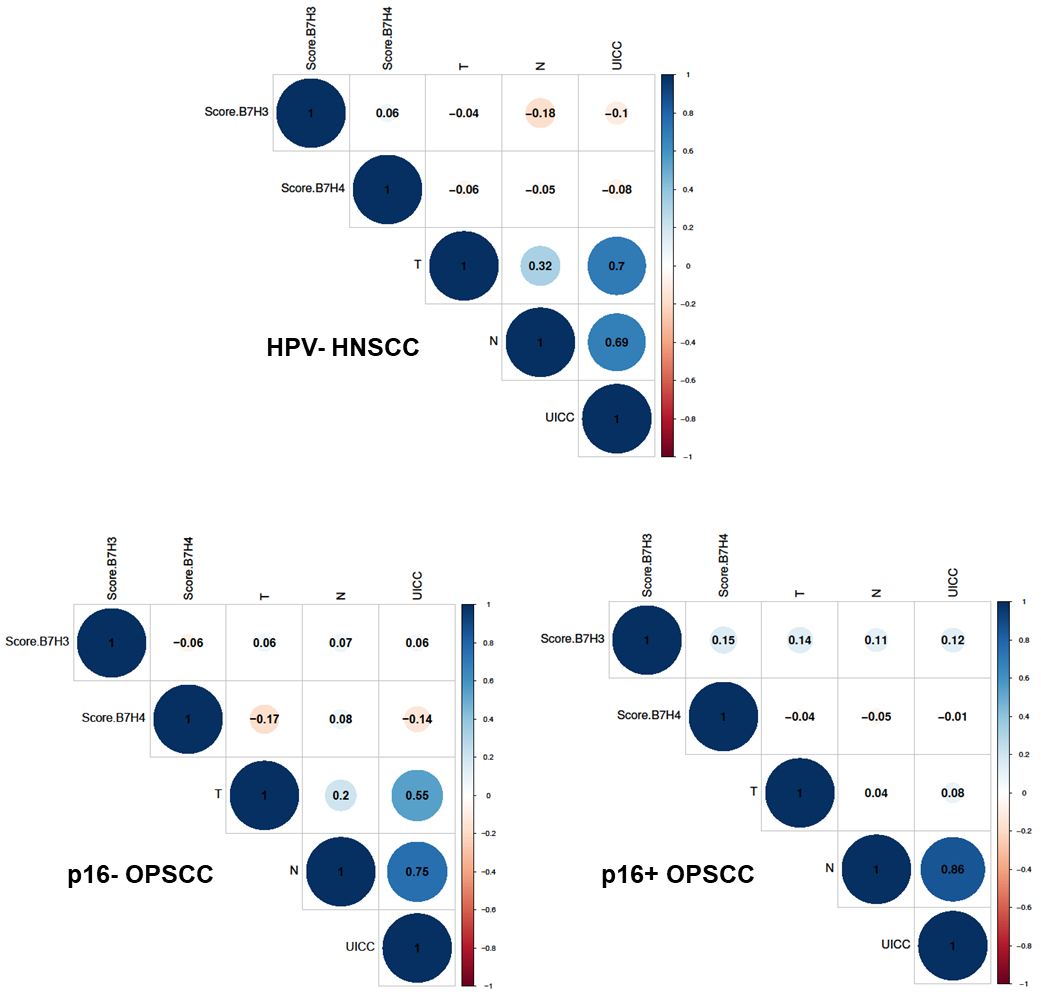


**Supplementary Figure 2. Correlation analyses.** Correlation analyses of B7-H3 and B7-H4 expression scores with each other and with T-, N- and UICC-stage (7th edition) in HPV- HNSCC and in OPSCC in dependence of the p16 status. As expected, T and N-stage show a clear correlation with UICC-stage (7th edition) in HPV- HNSCC and p16- OPSCC and N-stage with UICC-stage in p16+ OPSCC. B7-H3 and B7-H4 do not demonstrate a clear correlation with each other or T-, or N-stage. Note that faint assoziations are observed between B7-H3 and N-status in HPV- HNSCC and B7-H4 and T-status in p16- OPSCC.


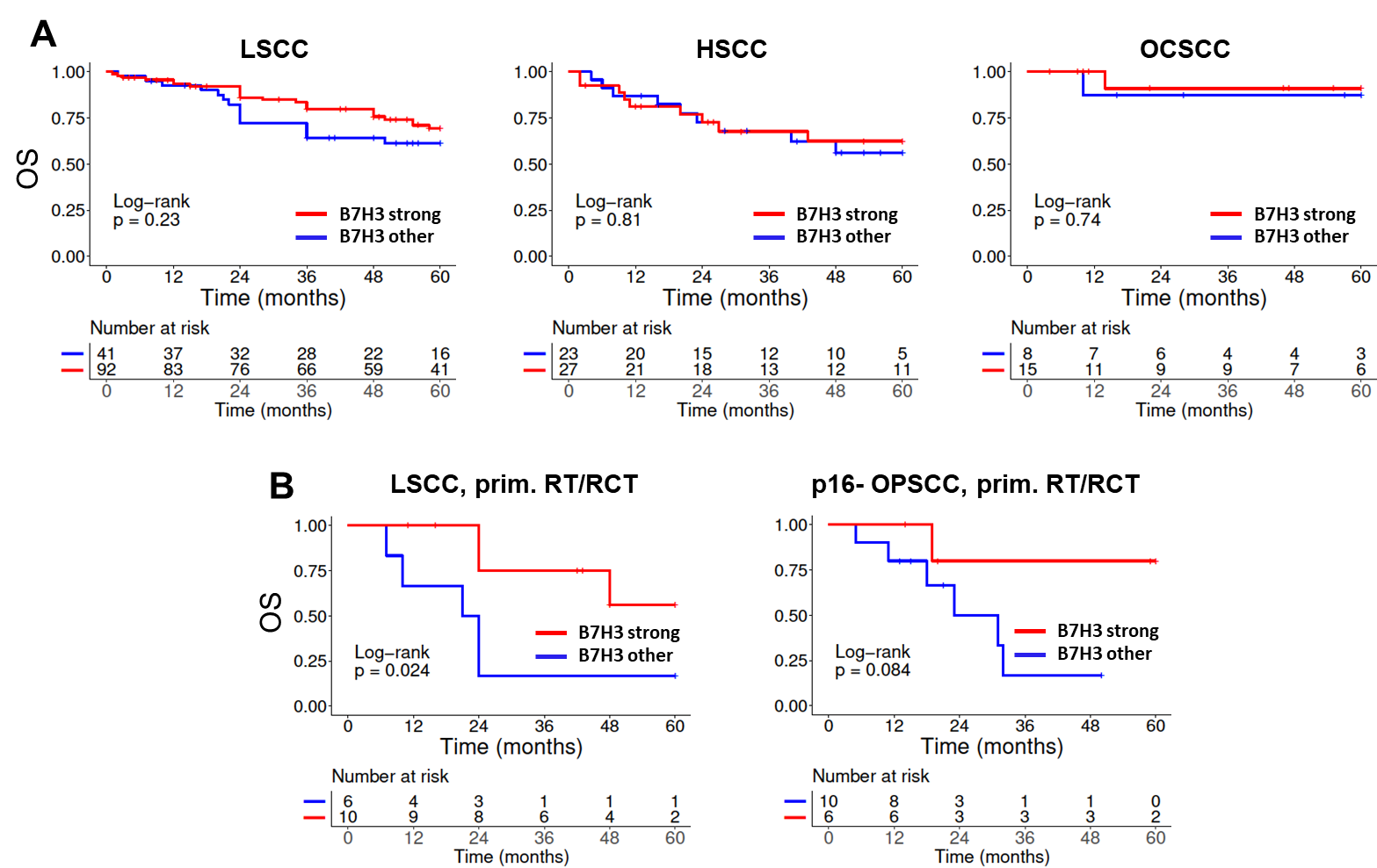


**Supplementary Figure 3. Association of overall survival and B7-H3 expression in the indicated subgroups.** B7-H3 expression was categorized as either strong or other, which includes all samples scored as negative, weak or moderate.


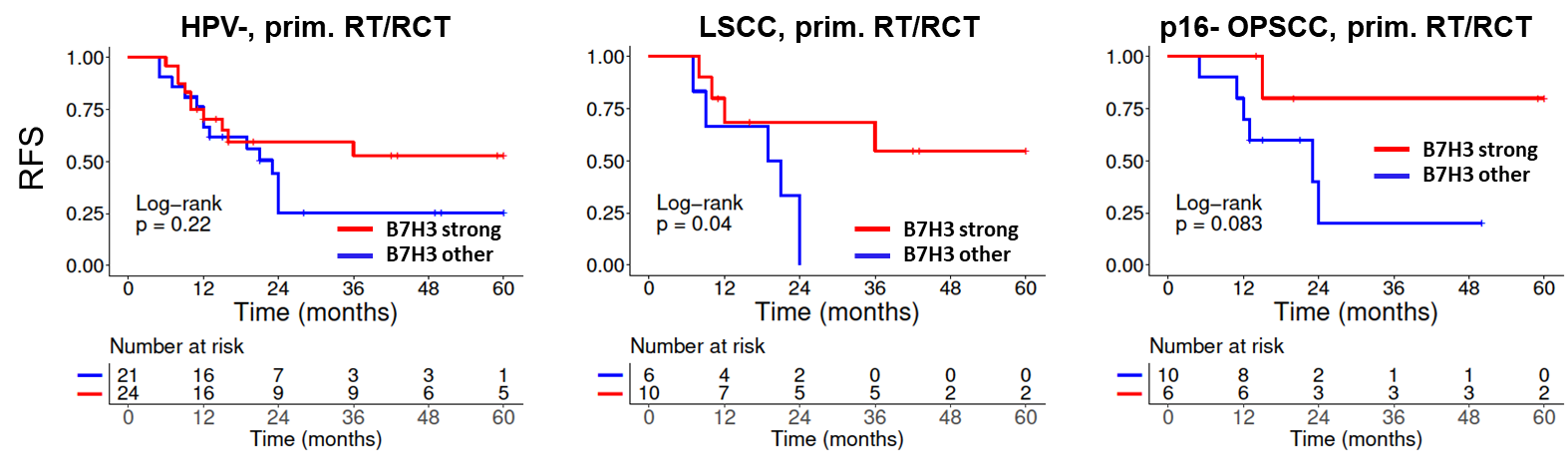


**Supplementary Figure 4. Association of recurrence free survival and B7-H3 expression in the indicated patients groups treated with primary RT/RCT**. B7-H3 expression was categorized as either strong or other, which includes all samples scored as negative, weak or moderate.
